# A Host Small GTP-binding Protein ARL8 Plays Crucial Roles in Tobamovirus RNA Replication

**DOI:** 10.1371/journal.ppat.1002409

**Published:** 2011-12-08

**Authors:** Masaki Nishikiori, Masashi Mori, Koji Dohi, Hideyasu Okamura, Etsuko Katoh, Satoshi Naito, Tetsuo Meshi, Masayuki Ishikawa

**Affiliations:** 1 Division of Plant Sciences, National Institute of Agrobiological Sciences, Tsukuba, Ibaraki, Japan; 2 Research Institute for Bioresources and Biotechnology, Ishikawa Prefectural University, Nonoichi, Ishikawa, Japan; 3 Graduate School of Agriculture, Hokkaido University, Sapporo, Hokkaido, Japan; University of Kentucky, United States of America

## Abstract

*Tomato mosaic virus* (ToMV), like other eukaryotic positive-strand RNA viruses, replicates its genomic RNA in replication complexes formed on intracellular membranes. Previous studies showed that a host seven-pass transmembrane protein TOM1 is necessary for efficient ToMV multiplication. Here, we show that a small GTP-binding protein ARL8, along with TOM1, is co-purified with a FLAG epitope-tagged ToMV 180K replication protein from solubilized membranes of ToMV-infected tobacco (*Nicotiana tabacum*) cells. When solubilized membranes of ToMV-infected tobacco cells that expressed FLAG-tagged ARL8 were subjected to immunopurification with anti-FLAG antibody, ToMV 130K and 180K replication proteins and TOM1 were co-purified and the purified fraction showed RNA-dependent RNA polymerase activity that transcribed ToMV RNA. From uninfected cells, TOM1 co-purified with FLAG-tagged ARL8 less efficiently, suggesting that a complex containing ToMV replication proteins, TOM1, and ARL8 are formed on membranes in infected cells. In *Arabidopsis thaliana*, *ARL8* consists of four family members. Simultaneous mutations in two specific *ARL8* genes completely inhibited tobamovirus multiplication. In an *in vitro* ToMV RNA translation-replication system, the lack of either TOM1 or ARL8 proteins inhibited the production of replicative-form RNA, indicating that TOM1 and ARL8 are required for efficient negative-strand RNA synthesis. When ToMV 130K protein was co-expressed with TOM1 and ARL8 in yeast, RNA 5′-capping activity was detected in the membrane fraction. This activity was undetectable or very weak when the 130K protein was expressed alone or with either TOM1 or ARL8. Taken together, these results suggest that TOM1 and ARL8 are components of ToMV RNA replication complexes and play crucial roles in a process toward activation of the replication proteins' RNA synthesizing and capping functions.

## Author Summary

Many important pathogens of plants, animals, and humans are positive-strand RNA viruses. They replicate via complementary RNA in replication complexes formed on host intracellular membranes. In the replication process, not only viral replication proteins but also host factors play important roles. Although many host factors whose knockdown affects the multiplication of positive-strand RNA viruses have been identified, the function of each host factor in virus multiplication is only poorly understood in most instances. In this paper, we show that a host small GTP-binding protein ARL8 is required for the multiplication of *Tomato mosaic virus* (ToMV), and that it forms a complex with ToMV replication proteins and another essential host factor TOM1 that is a seven-pass transmembrane protein. We further demonstrate that the replication proteins acquire the ability to synthesize negative-strand ToMV RNA and RNA 5′ cap only in the presence of both TOM1 and ARL8. The replication proteins of ToMV are multifunctional proteins that participate in RNA replication on membranes and RNA silencing suppression in the cytosol. Our results suggest that ToMV replication proteins are programmed to express their replication-related activities only on membranes through interactions with these host membrane proteins.

## Introduction

Many animal viruses of medical and veterinary importance such as *Poliovirus* and *Hepatitis C virus*, and most plant viruses, including *Tobacco mosaic virus* (TMV), *Brome mosaic virus* (BMV) and *Tomato bushy stunt virus* (TBSV) are positive-strand RNA viruses. These viruses have single-stranded, messenger-sense RNA genomes in virions. After infection, their genomic RNAs are released into the cytoplasm of host cells and are translated to produce viral proteins including those that are required for RNA replication (hereafter, replication proteins). The replication proteins recruit their genomic RNAs onto intracellular membranes and synthesize complementary, negative-strand RNAs. The negative-strand RNAs are sequestered with the replication proteins in membranous compartments that are isolated from the cytosol, and are used as templates to synthesize positive-strand RNA (genomic and, for certain viruses, subgenomic RNAs), which are released into the cytosol [1]. The membrane-bound complexes that synthesize viral positive-strand RNAs are called ‘replication complexes’.

The multiplication of positive-strand RNA viruses depends not only on viral replication proteins but also on host factors. To date, a large number of such host factors has been identified [2]–[6], however, their roles in the viral RNA replication are revealed only for limited instances. For example, molecular chaperones, heat shock protein 70 (HSP70), HSP40, HSP90, and cyclophilin B, are required for efficient replication of BMV, *Flock house virus*, TBSV, and/or *Hepatitis C virus*, presumably by modifying the structure of viral replication proteins [7]–[11]. Eukaryotic translation elongation factor 1A (eEF1A) and glyceraldehyde-3-phosphate dehydrogenase that can bind to positive-strand and negative-strand TBSV RNAs are recruited to TBSV replication complexes and facilitate negative-strand and positive-strand RNA synthesis, respectively [12], [13]. The reticulon homology proteins interact with BMV 1a replication protein and play important roles in the formation of both spherular and double-membrane replication compartments by regulating membrane curvature [14]. The endosomal sorting complexes required for transport (ESCRT) proteins also participate in the formation of TBSV replication compartments, but, unlike reticulon homology proteins, they are not present in the mature TBSV replication complexes [15]. Lipid composition of the membranes is also important for RNA replication of positive-strand RNA viruses [16]–[19]. The 3A proteins of *Poliovirus* and *Coxsackievirus* B3 (CVB3: a picornavirus) bind to GBF1, a guanine nucleotide exchange factor for a small GTP-binding protein ARF1, and modulates the function of GBF1-ARF1 to preferentially recruit phosphatidylinositol-4-kinase IIIβ over other effectors of ARF1 and to facilitate the formation of phosphatidylinositol-4-phosphate (PI4P) lipid-enriched organelles, which are the essential binding site for 3D polymerase [20]. Facilitation of viral RNA replication by modulation of lipid biosynthesis by viral proteins is also reported for other viruses [21]–[23].

The genus *Tobamovirus* includes TMV, *Tomato mosaic virus* (ToMV), *Youcai mosaic virus* (this virus is identical to TMV-Cg and, in this report, is referred to as TMV-Cg for consistency with our previous publications), and other related viruses. The genome of a tobamovirus is a non-segmented, single-stranded, 5′-capped RNA of 6.4 kilobases that encodes a replication protein of approximately 130 kDa (130K protein) and its read-through product of 180 kDa (180K protein), a cell-to-cell movement protein, and a coat protein (CP). The 130K protein has a methyltransferase-like domain that is involved in 5′ capping of progeny RNAs and a helicase-like domain, and the read-through region of the 180K protein contains a polymerase-like domain [24]. The 130K and 180K proteins are multifunctional proteins that participate in RNA replication and RNA silencing suppression [25]. A fraction of the 130K and 180K proteins co-translationally bind to the genomic RNA to form a ribonucleoprotein complex, termed pre-membrane-targeting complex (PMTC), which is targeted to membrane surfaces to form viral replication complexes. The negative-strand RNA is synthesized only after PMTC binds to membranes and kept sequestered in the replication complex [26], [27]. Another fraction of the 130K protein that stays in the cytosol (in a non-membrane-bound form) participates in RNA silencing suppression by binding to double-stranded small RNAs [28], [29]. If negative-strand RNA is synthesized in or leaks into the cytosol, it could be a source of double-stranded RNA that would trigger RNA silencing against the viral genome [15], [30]. Thus, functional specialization of the replication proteins into two alternative forms is important for the virus to multiply successfully, and should be strictly regulated by as yet undefined mechanisms.

In tobamovirus multiplication, several host factors including eEF1A, a subunit of eukaryotic translation initiation factor 3, TOM1, and TOM2A have been suggested to participate [31]–[35]. *TOM1* and *TOM2A* encode seven-pass and four-pass transmembrane proteins, respectively. In *A. thaliana*, simultaneous inactivation of *TOM1* and its paralog *TOM3* results in complete inhibition of multiplication of both TMV-Cg and ToMV [36], [37], whereas knockout of *TOM2A* and its related genes results in incomplete inhibition of TMV-Cg multiplication but had little effect on ToMV multiplication [38]. TOM1 interacts with tobamoviral helicase domain polypeptides and TOM2A [33], [34], [37], and is thought to help tether tobamovirus replication proteins on membranes. To understand the roles of these host factors in tobamovirus multiplication and to further identify novel host factor genes, especially those that are difficult to identify through genetic approaches, e.g., genes that are functionally redundant and that are essential for host viability, we have developed experimental systems that enable biochemical analyses of tobamovirus RNA replication. These include a translation and replication system for tobamovirus RNA in evacuolated plant cell extract (BYL) [39] and a method to prepare and purify active tobamovirus RNA-dependent RNA polymerase (RdRp) [27]. Our previous study showed that the purified ToMV RdRp preparation contained viral replication proteins, TOM1, TOM2A, HSP70, eEF1A, and an unidentified protein of approximately 20 kDa [27]. Here, we identify the 20-kDa protein and show that the protein and TOM1 play crucial roles in the process toward activation of the replication proteins' essential functions.

## Results

### Association of a small GTP-binding protein ARL8 with membrane-bound tobamovirus replication proteins

**Figure 1:**
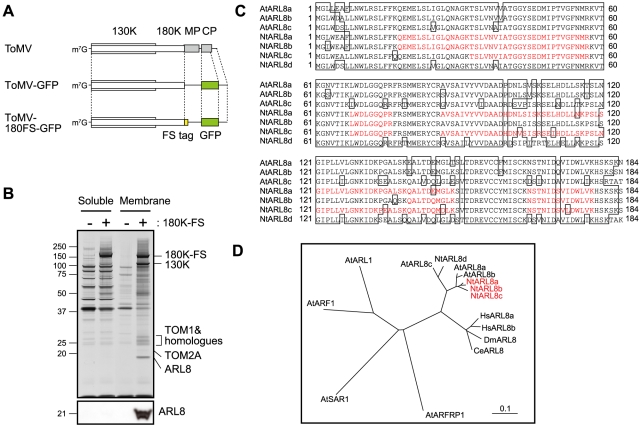
Analysis of proteins co-purified with the 180K-FS protein. (A) Schematic representation of ToMV, ToMV-GFP, and ToMV-180FS-GFP. (B) Silver staining of proteins contained in the FLAG-purified fractions “Copyright © American Society for Microbiology, Journal of Virology, 80, 2006, 8459-8468, doi:10.1128/JVI.00545-06”. Soluble and membrane fractions of ToMV-GFP- or ToMV-180FS-GFP-infected and evacuolated tobacco BY-2 protoplasts were prepared, treated with LPC, clarified by centrifugation, and subjected to affinity purification with monoclonal anti-FLAG antibody. Co-purified proteins were separated by SDS-12% PAGE and silver-stained (upper panel). The positions of protein markers are shown on the left with their molecular weights (x10^−3^). Proteins identified by LC-MS/MS are shown on the right. The bottom panel shows an immunoblot of the fractions detected with anti-ARL8 antibodies. (C) Alignment of deduced amino acid sequences of *A. thaliana* (At) and *N. tabacum* (Nt) ARL8 proteins. The sequence data of the AtARL8 proteins were obtained from the Arabidopsis Information Resource (http://www.arabidopsis.org). Peptides identified by LC-MS/MS are indicated by red letters. (D) Phylogenetic tree of ARF family proteins. An unrooted tree was constructed using ClustalW with standard parameters and drawn using TreeView. Scale bar represents 0.1-amino acid substitutions per site. Abbreviations: At, *Arabidopsis thaliana*; Nt, *Nicotiana tabacum*; Ce, *Caenorhabditis elegans*; Dm, *Drosophila melanogaster*; Hs, *Homo sapiens*.

In a previous study [27], we generated a transgenic tobacco (*Nicotiana tabacum*) BY-2 cell line in which infection of ToMV-180FS-GFP that encodes FLAG-StrepII-tagged 180K (180K-FS) (Figure 1A) could be induced by treatment with estradiol from a cDNA cassette integrated in the host genome. We prepared membrane fractions from ToMV-180FS-GFP-infected and evacuolated protoplasts, solubilized the membranes with a detergent lysophosphatidylcholine (LPC), and then purified the 180K-FS protein using an anti-FLAG antibody. The purified fraction contained viral 130K protein, host TOM1, TOM2A, HSP70, EF1A, and an unidentified protein of approximately 20 kDa ([27] and Figure 1B).

We analyzed the 20-kDa protein using liquid chromatography-tandem mass spectrometry (LC-MS/MS) and found that it was a small GTP-binding protein, NtARL8. NtARL8 co-purified with the 180K-FS protein from the solubilized membranes but not from the 15,000 x *g* supernatant of the extract of ToMV-180FS-GFP-infected and evacuolated protoplasts (Figure 1B, lower panel). By the degenerate PCR and RACE methods, we identified four *ARL8*-related genes from *N. tabacum* cv. Samsun and found by re-analyzing the LC-MS/MS data that the proteins from at least three of these genes, *NtARL8a*, *NtARL8b*, and *NtARL8c*, were co-purified with ToMV 180K-FS (Figure 1C; Genbank Accession Nos. AB644217-AB644220). ARL8 belongs to the ARF family of small GTP-binding proteins that include ARF and SAR proteins. ARF family proteins have an N-terminal amphipathic helix that participates in tight binding of the protein to membranes. *ARL8* family genes are conserved in plants and animals but not in yeast *S. cerevisiae* (Figure 1D) [40]–[42]. Human, *Drosophila melanogaster*, and *Caenorhabditis elegans* ARL8 proteins mainly localize to lysosomes and *C. elegans* ARL8 mediates transport of endocytosed macromolecules to lysosomes by facilitating late endosome-lysosome fusion [43]–[45]. *Arabidopsis* ARL8 proteins localize on vacuolar and other organellar membranes [46]. This is intriguing because ToMV RNA replication takes place on membranes and TOM1 is also localized on vacuolar and other organellar membranes.

### Interaction among tobamovirus replication proteins, TOM1, and ARL8

**Figure 2:**
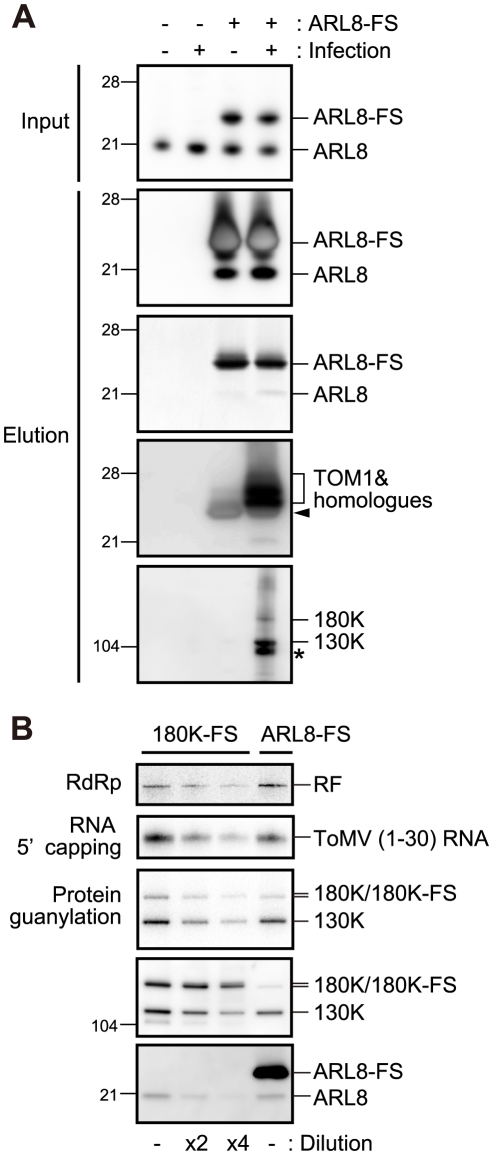
Affinity purification of ARL8-FS from uninfected and ToMV-infected BY-2 cells. (A) Copurification of TOM1, 130K and 180K proteins with NtARL8a-FS. The P15 membrane fractions were prepared from the extracts of NtARL8a-FS-expressing BY-2 cells that had or had not been infected with ToMV-GFP. NtARL8a-FS was solubilized with LPC and immunopurified with the anti-FLAG antibody [27]. NtARL8a-FS and endogenous ARL8 proteins were detected by immunoblotting and Coomassie brilliant blue-staining of the blotted membrane (second and third panels from the top, respectively). TOM1 and ToMV 130K and 180K replication proteins were detected by immunoblotting. Control experiments with BY-2 cells that did not express NtARL8a-FS were performed in parallel. The arrowhead shows signals corresponding to the crossreaction of anti-TOM1 antibodies with NtARL8a-FS. The asterisk indicates a degradation product of the 130K/180K proteins. The positions of protein markers are shown on the left with their molecular weights (x10^−3^). (B) Copurification of ToMV RdRp and capping activities with ARL8-FS. The P15 membrane fractions were prepared from ARL8-FS-expressing and ToMV-GFP-infected BY-2 cells or from ToMV-180FS-GFP-infected BY-2 cells that were evacuolated. The fractions were solubilized with LPC and immunopurified with the anti-FLAG antibody [27]. The FLAG-purified fractions were subjected to RdRp, RNA 5′ capping, and protein guanylation assays. The fractions were also subjected to immunoblot analysis to detect ToMV replication proteins and ARL8. Where specified, the fraction was diluted 2- or 4-fold. The RdRp reaction was performed in the presence of [α-^32^P]CTP using exogenously added ToMV RNA as a template as described previously [27], and analyzed by PAGE. RNA 5′ capping and protein guanylation reactions were performed and products were analyzed as described in the Materials and Methods. ^32^P-labeled bands were detected with an image analyzer (BAS 2500, Fujifilm). The positions corresponding to double-stranded ToMV RNA (RF), ToMV (1–30) RNA and 130K, 180K (180K-FS), and ARL8 (ARL8-FS) are indicated on the right.

Tobamovirus replication proteins associated with TOM1 and ARL8 ([27] and Figure 1B); however it was unclear whether a complex containing all of the three proteins is formed or the replication proteins form distinct complexes with TOM1 and ARL8. To assess these possibilities, we generated a transgenic BY-2 cell line in which C-terminally FLAG-StrepII-tagged NtARL8a (NtARL8a-FS) was stably expressed and infection of ToMV-GFP (Figure 1A) could be induced by treatment with estradiol. Membrane fractions were then prepared from NtARL8a-FS-expressing and uninfected or ToMV-GFP-infected protoplasts, and treated with LPC. From the solubilized fractions, NtARL8a-FS was affinity-purified. As expected, ToMV 130K and 180K proteins were co-purified with NtARL8a-FS from infected cells (Figure 2A, bottom panel). NtTOM1 was also co-purified with NtARL8a-FS from ToMV-infected cells. From uninfected cells, NtTOM1 was co-purified with NtARL8a-FS, but the amount was much lower than that from ToMV-infected cells (Figure 2A, fourth panel from the top). Endogenous NtARL8 was co-purified with NtARL8a-FS from uninfected and ToMV-infected cells at similar levels (Figure 2A, second panel from the top). These results suggest that a membrane-bound complex that contains ToMV replication proteins, NtTOM1, and NtARL8 is formed in infected cells.

We previously found that only a small fraction of the membrane-bound replication proteins in plant cells participates in RNA synthesis [27]. To investigate whether ARL8-bound replication proteins are active in synthesizing RNA, we prepared FLAG-purified fractions from solubilized membranes of NtARL8a-FS-expressing and ToMV-GFP-infected BY-2 protoplasts and ToMV-180FS-GFP-infected protoplasts, and compared RdRp activity per 180K protein in these fractions (the 180K protein but not the 130K protein has the polymerase-like domain). For RdRp assay, radiolabeled ribonucleoside triphosphates and ToMV virion RNA were added because endogenous replication templates dissociate from the replication proteins by solubilization with LPC [27]. A larger amount of the 180K protein was found in the ToMV-180FS-purified fraction (Figure 2B, leftmost lane) than in the NtARL8a-FS-purified fraction (Figure 2B, rightmost lane), whereas a similar level of RdRp activity was detected in these two lanes. This suggests that ToMV 180K protein that associates with NtARL8 is actively involved in RNA synthesis.

**Figure 3:**
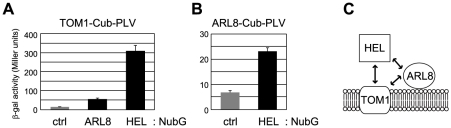
Interactions between TOM1, ARL8, and the helicase domain polypeptide (HEL) of ToMV replication proteins. (A) Split-ubiquitin assay using TOM1-Cub-PLV-expressing yeast. The graph shows β-galactosidase activity in the yeast reporter strain L40 coexpressing TOM1-Cub-PLV and either NubG-ALG5 (ctrl) [47], ARL8-NubG (ARL8), or NubG-HEL (HEL). ALG5 is an unrelated yeast protein used as a negative control. (B) Split-ubiquitin assay using ARL8-Cub-PLV-expressing yeast. The graph shows β-galactosidase activity in L40 yeast coexpressing ARL8-Cub-PLV and either NubG-ALG5 (ctrl) or NubG-HEL (HEL). Averages and standard deviations of β-galactosidase activity (Miller units) for three or four independent yeast transformants are indicated in panels A and B. (C) A model of interaction between TOM1, ARL8, and ToMV HEL polypeptide.

We next performed pairwise split-ubiquitin-based yeast two-hybrid experiments, which can be used to detect interactions between a soluble protein and a membrane protein as well as between two membrane proteins [47], for tobamovirus replication proteins, TOM1, and ARL8. The results suggested that AtARL8a interacts with both AtTOM1 and a polypeptide corresponding to the helicase domain of ToMV replication proteins (amino acid residues 648-1116 of ToMV 130K protein, Figure 3A, 3B). Consistent with our previous results using the Sos recruitment system [34], [37], the ToMV helicase domain polypeptide appeared to interact with AtTOM1 (Figure 3A). The signals for ARL8 interactions with TOM1 and ToMV helicase domain were 6 and 13 times weaker than the TOM1-helicase domain interaction signal. We could not detect an interaction between TOM1 or ARL8 and the other regions of tobamovirus replication proteins by the split-ubiquitin or Sos recruitment systems (data not shown).

### ARL8 is necessary for tobamovirus multiplication

Because ToMV 180K protein, which shows RdRp activity, preferentially associates with NtARL8 (Figure 2B), ARL8 may positively regulate tobamovirus multiplication. To explore this further, we took advantage of *A. thaliana* T-DNA and transposon insertion mutants. The *A. thaliana* genome encodes four *ARL8*-related genes, designated *AtARL8a-d* [41], [42], [48] (Figure 1C and 1D). Quantitative RT-PCR analysis showed that mRNA levels of *AtARL8a* (At3g49870), *AtARL8c* (At5g37680), and *AtARL8d* (At3g49860) are about 89%, 3%, and 0.5%, respectively, of that of *AtARL8b* (At5g67560). *AtARL8d* may be a pseudogene because it lacks an N-terminal part that is conserved among the other ARL8 proteins.

**Figure 4:**
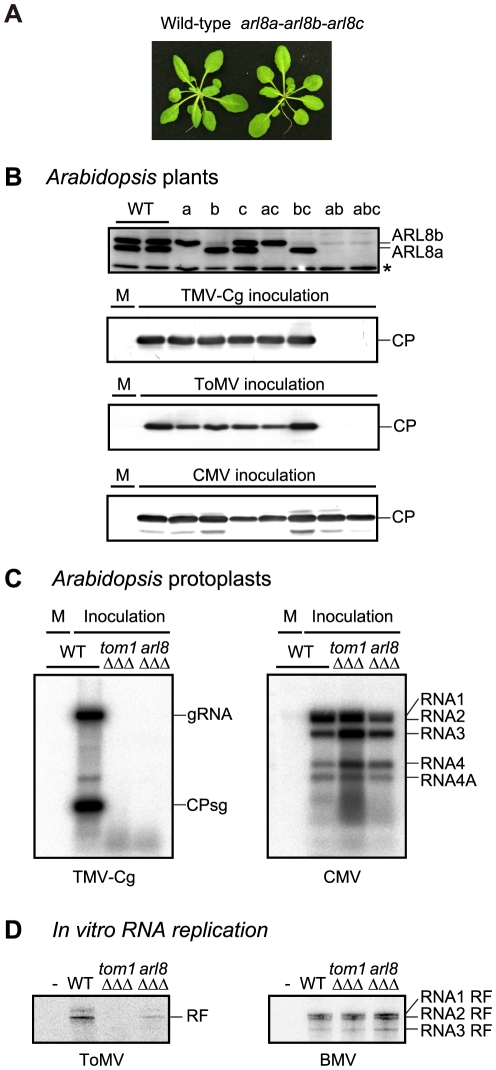
Effects of single and multiple mutations in *A. thaliana ARL8* family members on virus multiplication. (A) Wild-type (WT) and *arl8* triple mutant plants. The photograph was taken 5 weeks after sowing. (B) Effects of *arl8* mutations on the multiplication of TMV-Cg, ToMV, and CMV. Wild-type and *arl8a*, *arl8b*, and/or *arl8c* single, double, and triple mutant *A. thaliana* plants were inoculated with TMV-Cg, ToMV or CMV, as described previously [36]. Inoculated leaves were harvested 2 days after inoculation (for TMV-Cg and CMV) or 7 days after inoculation (for ToMV), and the CPs were detected by immunoblotting using a 13% acrylamide, 0.35% bisacrylamide gel. ARL8a and ARL8b proteins were also detected (top panel). The asterisk denotes a background signal derived from cross-reactivity of the anti-ARL8 antiserum to a cellular protein, which served as loading controls. M indicates mock inoculation. (C) Effects of *tom1 tom3 thh1* and *arl8a arl8b arl8* triple mutations on the multiplication of TMV-Cg (left panel) and CMV (right panel) in *A. thaliana* protoplasts. Protoplasts with each genotype were isolated from suspension-cultured cells, and inoculated with TMV-Cg and CMV RNA by electroporation [70]. Total RNA was isolated from the protoplasts 20 h after inoculation, and the accumulation of TMV-Cg and CMV RNA was analyzed by Northern blotting as described previously [70]. (D) Effect of the loss of TOM1 and ARL8 on the production of viral RF RNA in the *in vitro* translation-replication system. A ToMV RNA derivative TL180SF [26] and BMV virion RNAs were translated in mdBYL and ToMV PMTC were purified by 100,000 x *g* centrifugation and further with an anti-FLAG antibody to avoid contamination of soluble ARL8. For BMV, ribonucleoprotein complexes were purified by 100,000 x *g* centrifugation. The purified ribonucleoprotein fractions were mixed with membranes derived from protoplasts of suspension-cultured wild-type, *tom1* or *arl8* triple mutant *A. thaliana* cells. After the RdRp reaction, RNA samples were prepared, treated with a single-strand-specific S1 nuclease, and then analyzed by PAGE and autoradiography.

We obtained *A. thaliana* T-DNA or transposon insertion mutants *arl8a-1*, *arl8b-1*, and *arl8c-1*, and constructed all possible double and triple mutants. None of these *arl8* mutations had any apparent effect on plant growth or morphology (Figure 4A). For the *arl8b-1* single, *arl8a-1 arl8b-1* double or *arl8a-1 arl8b-1 arl8c-1* triple mutants, a faint band was detected at the position of the AtARL8b protein on the immunoblot (Figure 4B, top panel). Because a transposon fragment is inserted in one of introns in the *arl8b-1* mutant, a small amount of wild-type *ARL8b* mRNA might be produced via splicing that correctly links the exons. These mutant plants were inoculated with TMV-Cg and ToMV, and CP accumulation was examined 2 days and 7 days after inoculation, respectively. The CPs were not detected in the inoculated leaves of *arl8a-1 arl8b-1* double or *arl8a-1 arl8b-1 arl8c-1* triple mutants (Figure 4B), whereas in the other mutants, the CPs accumulated to a level similar to that in wild-type plants. That no single mutation in the *ARL8* family members showed any detectable phenotype on tobamovirus multiplication may explain why we previously failed to identify *arl8* alleles by screening randomly mutagenized *A. thaliana* plants. The CP of *Cucumber mosaic virus* (CMV) accumulated to wild-type levels in all of these *arl8* mutants (Figure 4B, bottom panel). These results indicate that ARL8 plays a specific and essential role in tobamovirus multiplication *in planta*.

To examine whether ARL8 proteins are required for tobamovirus multiplication in single cells, we established *A. thaliana* suspension-cultured cells from wild-type, *tom1-2 tom3-1 thh1-1* (triple) [36], and *arl8a-1 arl8b-1 arl8c-1* (triple) mutants. Protoplasts were prepared from these cells and inoculated with TMV-Cg and CMV RNAs. TMV-Cg RNA accumulated in wild-type protoplasts at 20 h post-inoculation, but was not detected in *tom1-2 tom3-1 thh1-1* or *arl8a-1 arl8b-1 arl8c-1* mutant protoplasts (Figure 4C, left panel). In these mutant protoplasts, CMV RNA accumulated to a level similar to that in wild-type protoplasts (Figure 4C, right panel). These results indicate that TOM1 and ARL8 are necessary for intracellular tobamovirus multiplication.

### Deficiency in either TOM1 or ARL8 inhibits the production of ToMV negative-strand RNA *in vitro*

In protoplast inoculation assays, viral replication products become detectable only after multiple rounds of RNA replication. Thus, inability of detection of progeny RNA in inoculated protoplasts does not necessarily indicate that the cells cannot support negative-stand RNA synthesis. To test the requirement of TOM1 and ARL8 for ToMV negative-strand RNA synthesis, we used an *in vitro* ToMV RNA translation and replication system [26], [39]. In this system, ToMV RNA is first translated in membrane-depleted evacuolated tobacco BY-2 protoplast extracts (mdBYL). By this reaction, the replication proteins are synthesized and the PMTC, a complex containing the ToMV RNA and replication proteins, is formed. The negative-strand RNA synthesis does not occur at this stage, but it does after the PMTC is incubated with membranes prepared from evacuolated plant protoplasts. In this system, negative-strand RNA synthesis, represented by the production of the double-stranded RNA called replicative form (RF) RNA, is detectable without multiple rounds of the replication cycle [26].

We translated ToMV RNA that encodes the 180K-FS protein in mdBYL and immunopurified the PMTC using the FLAG tag. The mdBYL contained small amounts of NtARL8 protein, but NtARL8 was not detectable in the purified PMTC fraction. NtTOM1 was also undetectable in the PMTC fraction. The PMTC was incubated with membranes prepared from wild-type, *tom1-2 tom3-1 thh1-1*, and *arl8a-1 arl8b-1 arl8c-1* triple mutant *A. thaliana* suspension-cultured cells, and then with substrates for RNA synthesis containing [α-^32^P]CTP. After the reaction, total RNA was purified, treated with S1 nuclease, and separated by PAGE. This analysis showed that ToMV RF RNA, indicative of negative-RNA synthesis, was produced in the mixture to which membranes from wild-type cells were added (Figure 4D). In contrast, in samples to which membranes from the *tom1-2 tom3-1 thh1-1* or *arl8a-1 arl8b-1 arl8c-1* triple mutants were added, the production of ToMV RF RNA was less than 1% and 20% of that for wild-type, respectively (Figure 4D). Membranes from the mutants supported RF RNA production of BMV as did the wild-type membranes (Figure 4D), confirming their integrity. Although it is unclear whether the low-level production of ToMV RF RNA with *arl8* triple mutant membranes is due to the presence of a small amount of ARL8 proteins or whether ARL8 is not absolutely required for RF RNA production, these results suggest that TOM1 and ARL8 are essential for efficient ToMV negative-strand RNA synthesis.

### Co-expression of TOM1, but not ARL8, increases the relative amount of the membrane-bound tobamovirus replication proteins in yeast

To investigate the respective roles of TOM1 and ARL8 in tobamovirus multiplication, we used *S. cerevisiae*, which does not have genes homologous to *TOM1* or *ARL8* and is known to support the replication of some higher eukaryotic positive-strand RNA viruses [49]. In yeast, AtTOM1 and AtARL8a proteins and the ToMV helicase polypeptide could be expressed. However, expression of full-length 130K protein was not detected when the coding sequence for the protein was inserted downstream of a yeast promoter and introduced into yeast. We found that the original coding sequence for the ToMV 130K protein contains putative poly(A) addition-transcription termination signals [50]. Thus, we engineered the ToMV 130K-coding sequence by introducing synonymous mutations to remove the signals and to optimize codon usage for expression in yeast. When yeast carrying the modified cDNA for ToMV 130K protein under the control of the Cu^2+^-inducible *CUP1* promoter was cultured in conditions under which the promoter was fully induced (0.2 mM CuSO_4_), the 130K protein accumulated to a level detectable in total cellular proteins separated by SDS-PAGE and stained with Coomassie brilliant blue dye. Because this level of 130K protein per total protein was higher than that observed in ToMV-infected plant cells, yeasts carrying the *CUP1* promoter-driven modified 130K protein-coding sequences were cultured, unless otherwise specified, in the presence of a lower concentration (1 µM) of CuSO_4_ so that the expression level of the protein (per total protein) was comparable to that in ToMV-infected plant cells.

**Figure 5:**
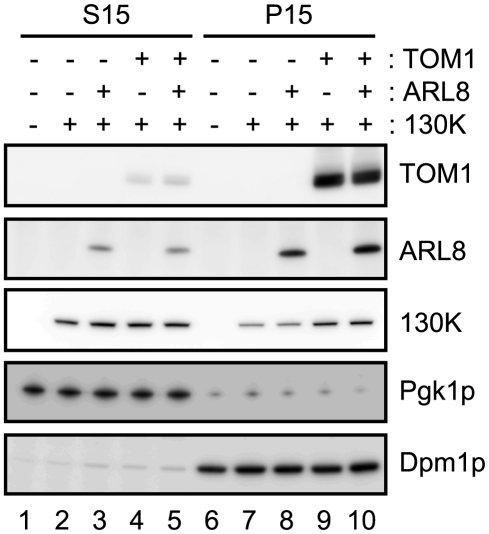
Effects of TOM1 and ARL8 coexpression on the membrane-association of ToMV 130K protein. The soluble (S15) and membrane (P15) fractions were prepared from yeasts expressing the indicated proteins and subjected to immunoblot analysis using antibodies against TOM1, ARL8, ToMV 130K, a yeast cytoplasmic protein Pgk1p, and an ER-localized protein Dpm1p. S15 samples prepared from 0.12 OD_600_ units of yeast cells or P15 samples prepared from 0.6 OD_600_ units of yeast cells were applied in each lane.

Using the expression system in yeast, we examined whether coexpression of TOM1 and/or ARL8 affects the subcellular fractionation pattern of the 130K protein. We fractionated extracts of yeast expressing the 130K protein alone or with AtTOM1 and/or AtARL8a by centrifugation (15000 x *g*) into supernatant (S15) and precipitate (P15) fractions. Immunoblot analysis showed that the amount of the 130K protein in the membrane-containing P15 fraction from AtTOM1-coexpressing yeast was larger than that from yeast expressing the 130K protein alone or the 130K plus AtARL8a proteins, whereas in the S15 fraction, the amount of the 130K protein was not detectably affected by coexpression of AtTOM1 or AtARL8a (Figure 5). Consistent results were obtained using the membrane-flotation assay [28]. These results suggest that TOM1 increases the relative amount of the membrane-bound 130K protein.

### TOM1 and ARL8 cooperatively activate tobamovirus replication proteins' capping function

**Figure 6:**
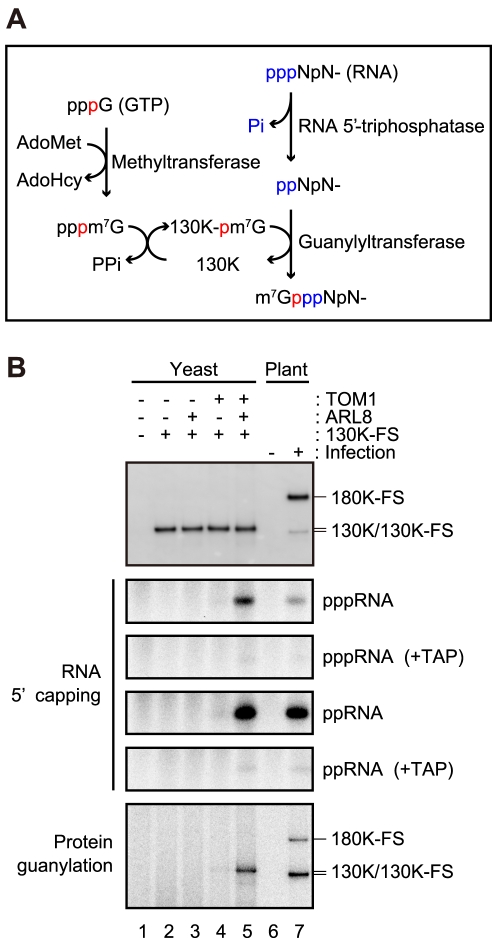
Effects of TOM1 and ARL8 coexpression on RNA 5′ capping functions of ToMV 130K protein. (A) A possible mechanism of RNA 5′ capping by tobamovirus replication proteins. The phosphate group at the α position of GTP is shown in red. The 5′ phosphate groups of the substrate RNA are shown in blue. See text for details. AdoHcy: *S*-Adenosyl-L-homocysteine. (B) Effects of the coexpression of TOM1 and ARL8 on RNA 5′ capping and guanylation of ToMV 130K protein expressed in yeast. P15 membrane fractions were prepared from the indicated yeast and BY-2 cells. The fractions were treated with LPC and subjected to immunopurification with anti-FLAG antibody [27]. The protein samples were prepared so that the concentration of the replication proteins was similar (samples for lanes 1 and 6 were prepared and analyzed in the same way as for those for lanes 2 and 7, respectively). The samples were incubated at 25°C for 60 min with [α-^32^P]GTP, AdoMet and uncapped ToMV (1–30) RNA with 5′-triphosphate or 5′-diphosphate. After the reaction, RNA was purified by phenol extraction and separated by 8 M urea-9% PAGE. Fractions of the purified RNA samples were treated with TAP and analyzed similarly. ^32^P-labeled RNA was visualized with an image analyzer (BAS2500, Fujifilm). The protein samples were also subjected to immunoblot analysis with anti-ToMV replication protein antibodies and protein guanylation assay as described in the Materials and Methods.

Membrane-bound tobamovirus 130K protein isolated from infected plant cells has activities to transfer a methyl group from *S*-adenosyl-L-methionine (AdoMet) to GTP, to form a covalent guanylate complex, and to transfer the guanylate moiety to the 5′-terminus of newly synthesized RNA to generate m^7^G(5′)ppp(5′)-capped RNA [51] (Figure 6A). We examined whether ToMV 130K protein, expressed alone or with TOM1 and/or ARL8, in yeast would also show this capping activity. Membrane-bound 130K protein with the FLAG-StrepII tag at the C-terminus (130K-FS) expressed in yeast with or without the plant factors were solubilized with LPC and immunopurified. As a reference, membrane-bound 180K-FS protein from ToMV-180FS-GFP-infected plant cells was also solubilized and purified in the same way (Figure 6B, top panel). The purified fractions were incubated with AdoMet, [α-^32^P]GTP, and uncapped (5′-triphosphate) RNA that corresponds to nucleotides 1–30 of ToMV RNA [hereafter ToMV (1–30) RNA; this RNA has only one guanylate residue at the first position from the 5′ terminus], and RNA was extracted and analyzed by PAGE. ^32^P-labeled RNA, indicating capped RNA, was detected in the 180K-FS-purified fraction from infected plant cells and in 130K-FS-purified fraction from yeast coexpressing AtTOM1 and AtARL8a (Figure 6B, second panel from the top, lane 5 and 7). The ^32^P-labeled bands disappeared after treatment with tobacco acid pyrophosphatase (TAP) (Figure 6B, third panel from the top), confirming that the signals indeed represent the 5′ cap. ^32^P-labeled RNA was not detected in the lanes for yeasts expressing 130K-FS alone or with either AtTOM1 or AtARL8a (Figure 6B, second panel from the top). These results suggest that TOM1 and ARL8 facilitate activation of the 5′ capping function of the 130K protein on yeast membranes. Consistent with this, the FLAG-purified fraction from the membranes of ToMV-infected, NtARL8a-FS-expressing BY-2 cells also showed RNA 5′ capping activity (Figure 2B).

In bamboo mosaic virus RNA capping, newly synthesized RNA that has a 5′-triphosphate terminus accepts m^7^GMP after removal of the γ-phosphate group from the 5′-terminus by RNA 5′-triphosphatase activity associated with the helicase domain of the replication protein (Figure 6A) [52], [53]. Thus, we examined whether the 130K-FS protein, expressed alone or with AtTOM1 and/or AtARL8a, in yeast can add a 5′ cap to ToMV (1–30) RNA with 5′-diphosphate. Like the result with ToMV (1–30) RNA with 5′-triphosphate, ^32^P-labeled (i.e., capped) RNA was detected in the 130K-FS-purified fraction from yeast coexpressing AtTOM1 and AtARL8a, but not in the fractions from yeasts expressing 130K-FS alone or with either AtTOM1 or AtARL8a (Figure 6B, fourth and fifth panel from the top, lane 5 and 7).

We further examined whether 130K-FS protein co-expressed with AtTOM1 and/or AtARL8a can be guanylated. Affinity-purified 130K-FS proteins from solubilized membranes of AtTOM1- and/or AtARL8-coexpressing yeasts were incubated with AdoMet and [α-^32^P]GTP, and proteins were analyzed by SDS-PAGE for the production of ^32^P-guanylated 130K-FS protein. A ^32^P-labeled band at the position of the 130K-FS protein was detected in the 130K-FS-purified fraction from yeast coexpressing AtTOM1 and AtARL8a. ^32^P-labeled bands were also detected at the positions of the 130K and 180K-FS proteins in 180K-FS-purified fraction from infected plant cells, as expected. The ^32^P-labeled band was only very weakly detected for yeast expressing 130K-FS with AtTOM1 (Figure 6B, bottom panel, lane 5 and 7). When 130K-FS-purified fractions were prepared from yeast that over-expressed 130K-FS alone in the presence of a high concentration (20 µM) of CuSO_4_, and incubated with AdoMet and [α-^32^P]GTP, ^32^P-labeled 130K-FS was faintly detected. However, RNA 5′ capping activity was not detected in the fraction (data not shown). Taken together, these results suggest that activation of the 130K protein’s capping functions depends on coexpression of TOM1 and ARL8.

## Discussion

### TOM1 and ARL8 are essential components of tobamovirus replication complexes

In this study, we identified a small GTP-binding protein ARL8 that co-purifies with ToMV 180K-FS that has been solubilized by LPC from membranes of ToMV-infected plant cells (Figure 1). ToMV replication proteins and TOM1 were also efficiently co-purified with ARL8-FS from solubilized membranes of ToMV-infected cells, while TOM1 was co-purified with ARL8-FS less efficiently from solubilized membranes from uninfected cells (Figure 2). Because ToMV RNA and its complementary RNA dissociate from the replication proteins by LPC treatment [27], it seems unlikely that the observed association is mediated by RNA. Consistently, a yeast two-hybrid assay suggested that the helicase domain fragment of ToMV replication proteins, TOM1, and ARL8 interact with each other (Figure 3). Together, these results suggest that ToMV replication proteins form a complex with TOM1 and ARL8 on membranes.

Additionally, the ARL8-FS-purified fraction from solubilized membranes of ToMV-infected plant cells showed higher RdRp activity per 180K protein than the 180K-FS-purified fraction from solubilized membranes of ToMV-FS-infected plant cells did (Figure 2), suggesting that (i) most of the membrane-bound 180K protein does not have RdRp activity and that (ii) a small fraction of membrane-bound 180K protein that is associated with ARL8 and probably TOM1 does have RdRp activity. Together with the finding that mutations in the *TOM1* or *ARL8* genes seriously affect tobamovirus multiplication (Figure 4), these results suggest that TOM1 and ARL8 are essential components of tobamovirus replication complex.

### Roles of TOM1 and ARL8 in tobamovirus RNA replication

To investigate the roles of TOM1 and ARL8 in tobamovirus RNA replication, we first used an *in vitro* ToMV RNA translation-replication system using a plant cell extract BYL, in combination with TOM1- or ARL8-deficient membranes from *A. thaliana* mutant cells. The results showed that, in the absence of TOM1 or ARL8, production of negative-strand RNA is severely inhibited, suggesting that RNA polymerase activity failed to be activated (Figure 4). With the second approach, using a heterologous expression system in yeast, we found that (i) TOM1 increases the relative amount of the membrane-bound tobamovirus replication proteins and (ii) TOM1 and ARL8 confer the guanylyltransferase activity to ToMV 130K protein (Figure 5 and Figure 6). It is unlikely that negative-strand RNA was synthesized but undetectable due to the instability of uncapped RNA in the first assay, because (i) mutants of ToMV and BMV that are defective in RNA 5′ capping functions can still produce negative-strand RNA (Figure S1 and [54]), and (ii) negative-strand RNA should be in a membranous compartment that uncapped RNA-degrading enzymes cannot enter [27]. Rather, the results suggest that both of these functions fail to be activated when either TOM1 or ARL8 is absent. If active tobamovirus replication complexes are formed through a process involving multiple successive steps, TOM1 and ARL8 would be required for one or more steps of tobamovirus replication complex formation before the replication proteins gain these enzymatic activities.

The replication proteins of tobamoviruses consist of three domains, the methyltransferase-like, helicase-like, and polymerase-like domains [24]. Because TOM1 and ARL8 interact at least with the helicase-like domain (Figure 3) and because the interaction leads to activation of both 5′ capping and polymerase functions (Figure 4 and Figure 6), changes in the helicase domain caused by binding of these host factors may induce conformational changes involving the other two domains. Alternatively, by analogy to ARF1 function in polio and other viruses [20], effectors associated with TOM1 and/or ARL8 may contribute to establishing a microenvironment on the membranes that is essential for the formation of the tobamovirus replication complexes. In support of this possibility, specific lipids are required for activation of the capping function of semliki forest virus nsP1 and polymerase function of flock house virus protein A, respectively [19], [55]. However, the fact that TOM1 and ARL8 facilitate the activation of tobamovirus replication protein's capping function in yeast, in which the effectors of ARL8 are supposed to be absent, support the possibility that the TOM1 and ARL8 proteins themselves are required for activation of the capping-related functions.

Eukaryotic cells have RNA silencing and innate immune systems that are triggered by double-strand RNA and that target positive-strand RNA viruses. Thus, for successful multiplication, positive-strand RNA viruses must avoid negative-strand RNA synthesis in the cytosol and tightly sequester negative-strand RNA replication intermediates in isolated, membranous compartments [56], [57]. Thus, it is reasonable that the replication proteins of many eukaryotic positive-strand RNA viruses have amino acid sequences or modifications that strongly facilitate membrane binding [58]–[60]. For tobamoviruses, while the replication complexes are formed in isolated, membranous compartments [27], a majority of the replication proteins (about 90%) stay in the cytosol of plant cells and play roles in suppression of RNA silencing and probably other host defense [25], [28], [61]–[64]. The host membrane protein-dependent activation of replication-related enzymatic activities may contribute to securing the functional differentiation of the replication proteins and avoiding ectopic synthesis of negative-strand RNA in the cytosol.

## Materials and Methods

### Plants and suspension-cultured plant cells

Transgenic *Nicotiana tabacum* BY-2 cell lines E182 and RT4, in which infection with GFP-expressing ToMV derivatives that encode nontagged and C-terminally FLAG-StrepII-tagged 180K proteins (ToMV-GFP and ToMV-180FS-GFP), respectively, can be induced by treatment with estradiol were prepared as described previously [27], [65]. Note that the C-terminally FLAG-StrepII-tagged ToMV 180K protein fully retains the ability to support ToMV RNA replication [26]. For expression of C-terminally FLAG-StrepII-tagged *N. tabacum* ARL8a (NtARL8a-FS) in BY-2 cells, a cDNA fragment including the NtARL8a-FS-coding region was amplified by the overlap extension PCR method from pNtARL8a (see below) and pTL180SF-SmaI [26] by using primers, #1 plus #2, and #3 plus #4 (Table S1). The *NtARL8a-FS* fragment was digested with StuI and EcoRI and then cloned between the StuI and EcoRI sites of pBICBP35 [66]. E182 cells were further transformed with an *Agrobacterium tumefaciens* EHA105 strain carrying pBICBP35-NtARL8aFS to establish a cell line named AF/E182. Maintenance of BY-2-derived cells and induction of ToMV infection were performed as described previously [27].

*A. thaliana arl8a-1* (GABI-Kat 395G08), *arl8b-1* (RIKEN 11-6303-1), and *arl8c-1* (GABI-Kat 218A04) mutants were obtained from GABI-Kat and RIKEN. T-DNA and transposon insertions were confirmed by using primers, #5 and #6 (for *arl8a-1*), #7 and #8 (for *arl8b-1*), and #9 and #10 (for *arl8c-1*). *Arabidopsis thaliana* suspension-cultured cells were established from callus cultures generated from seedlings of wild-type (ecotype Col-0), *tom1-2 tom3-1 thh1-1* [36], and *arl8a-1 arl8b-1 arl8*c*-1* mutants. *A. thaliana* suspension-cultured cells were maintained by subculturing in fresh BY-2 growth medium at a dilution of 1∶6∼12 every 7 days. Cells were used for experiments 3 days after subculturing.

### Identification of ARL8

Membrane-bound 180K-FS protein was solubilized and purified from ToMV-180FS-GFP-infected and evacuolated BY-2 protoplasts as described previously [27]. Co-purified proteins were separated by SDS-PAGE (NuPAGE 12% Bis-Tris gel: Invitrogen, Carlsbad, CA) and visualized by silver-staining (Wako, Osaka, Japan). Proteins in excised gel pieces were subjected to digestion with typsin, LC-MS/MS analysis, and MASCOT searching (this part was performed by APRO Life Science Institute, Tokushima, Japan). *ARL8*-related genes in *N. tabacum* (*NtARL8s*) were isolated by degenerate PCR using SMART cDNA (Clontech, Mountain View, CA) prepared from *N. tabacum* poly(A)^+^ RNA as templates. mRNA sequences of the *NtARL8* genes were determined by the 5′ and 3′ random amplified cDNA ends method using the SMART cDNA kit. *NtARL8a* cDNA was amplified by RT-PCR from *N. tabacum* total RNA using primers #11 and #12, and cloned into pCR2-TOPO (Invitrogen) to obtain pNtARL8a.

### Antibodies

An anti-ARL8 antiserum was prepared in rabbits using N-terminally truncated NtARL8a protein (amino acid residues 17–185) expressed in *E. coli* [67] as an antigen and was used for immunoblot analysis at 1∶1000 dilution. The antiserum recognized AtARL8a, AtARL8b, and AtARL8c (Figure 4B and data not shown). Rabbit antisera against TOM1 and ToMV 130K/180K proteins have been described previously [62]. Anti-Pgk1p and anti-Dpm1p were purchased from Invitrogen.

### Split-ubiquitin assay

A DNA fragment containing the *A. thaliana* ARL8a (AtARL8a: At3g49870)-coding region was amplified by RT-PCR from *A. thaliana* total RNA using the primers #13 and #14, and was cloned into pCR2-TOPO (Invitrogen) to obtain pAtARL8a. DNA fragments corresponding to the *ADH1* promoter and AtARL8a-coding regions were amplified from pADNS [68] and pAtARL8a by PCR using primers, #15 plus #16 and #17 plus #18, respectively, and then the two fragments were fused by the overlap extension PCR method. The fused fragment was digested with XhoI and used to replace the XhoI fragment of pRS305(Δwbp1-Cub-PLV) [47] to obtain a plasmid for ARL8-Cub-PLV expression. A DNA fragment corresponding to the AtARL8a-coding region was amplified from pAtARL8a by PCR using primers, #18 and #19. The fragment was digested with ClaI and XhoI and used to replace the ClaI-XhoI fragment of pTN-TOM1-NubG [33] to obtain a plasmid for AtARL8a-NubG expression. A DNA fragment corresponding to amino acid residues 648-1116 of ToMV 130K protein was obtained from a plasmid for 5′SOS-LHel expression [37] by NcoI and NotI digestion. The fragment with a synthetic NotI-PstI linker was inserted between the NcoI and PstI sites of pTN-NubG-TOM2A [33] to obtain a plasmid for NubG-HEL expression. NubG-ALG5 that was used as a negative control was described previously [47]. β-galactosidase assay was performed as described previously [33].

### Expression of ToMV 130K, ARL8 and TOM1 proteins in yeast

A protease-deficient yeast strain BJ5465 (*MATa ura3-52 trp1 leu2Δ1 his3Δ200 pep4::HIS3 prb1Δ1.6R can1*) [8] was used. The *CUP1* promoter and *ADH1* terminator sequences were amplified by PCR from pRS314(NubG-ALG5) [47] and pADNS using primers, #20 plus #21, and #22 plus #23, respectively. The PCR products were digested with [KasI plus SnaBI] and [SacI plus HindIII], respectively. These two fragments, a synthetic SnaBI-NotI-SacI linker (#24 and #25) and YCp22 plasmid that had been double-digested with KasI and HindIII were mixed and ligated to obtain YCp22-CUP. Synthesis of the optimized 130K protein coding fragment-containing plasmid was performed by GENEART (Regensburg, Germany)-TAKARA (Shiga, Japan; Accession number AB642602). The optimized fragment with and without the C-terminal FLAG-StrepII tag was cloned between the SnaBI and NotI sites of YCp22-CUP to obtain YCp22-CUP-130Kv2 and YCp22-CUP-130Kv2-FS, respectively. AtTOM1 and AtARL8a proteins were expressed from *S. cerevisiae ADH1* and *CYC1* promoter-driven gene cassettes on YEp vectors [69] (YEp195-ADH-TOM1 and YEp181-CYC-ARL8), respectively.

### Isolation of tobamovirus replication proteins from yeast and plant cells

Yeasts expressing ToMV 130K protein, AtTOM1 and/or AtARL8a were treated with lyticase (125 units per mL) in 1 M sorbitol-0.1 M potassium phosphate (pH 7.6) for 30 min at 30°C, and the resulting spheroplasts were washed twice with 1 M sorbitol [60]. Yeast spheroplasts were disrupted in hypotonic buffer [10 mM Tris-HCl (pH 8), 10 mM EDTA with one tablet of Complete Mini protease inhibitors EDTA-free (Roche Diagnostics GmbH, Mannheim, Germany) per 10 ml] with 50 strokes of a tight-fitting Dounce homogenizer, and the homogenates were centrifuged at 800 x *g* for 10 min at 4°C to remove nuclei and cell debris. Induction of ToMV infection in tobacco BY-2-derived cell lines, preparation of protoplasts and extracts from the cells, and isolation of the P15 membrane fractions were conducted as described previously [27], except that the hypotonic buffer was used for cell disruption in Figure 5 and 6.

### RNA 5′ capping and protein guanylation assays

Purification and solubilization of P15 membrane fractions from ToMV 130K-FS-expressing yeast cells and ToMV-180FS-GFP-infected BY-2 cells were performed as described previously [27], except that solubilization buffer [30 mM Hepes-KOH (pH 7.4), 150 mM NaCl, 1 mM MgOAc, 0.1 mM DTT, 0.2 mM ATP and UTP, 1% LPC (Wako), and one tablet of Complete mini protease inhibitor EDTA-free per 10 mL] was used. Uncapped RNA corresponding to the ToMV 5′ untranslated region (nucleotides 1-30) was synthesized with an AmpliScribe T7-FLASH kit (Epicentre, Madison, WI) using annealed oligonucleotides #26 and #27 as template. ToMV (1–30) RNA has only one guanylate residue at the 5′ end and 5′-triphosphate and 5′-diphosphate RNAs were generated by *in vitro* transcription in the presence of ATP, UTP, CTP and either GTP or GDP. The 180K protein- or 130K protein-purified fractions were mixed with AdoMet (100 µM), GTP (1 µM), [α-^32^P] GTP (10 µCi in 30 µL reaction mixture) and the uncapped RNA substrate (1 µg in 30 µL reaction mix), and incubated at 25°C for 1 h, followed by RNA purification by phenol extraction and alcohol precipitation. Treatment of the RNA with tobacco acid pyrophosphatase (Nippongene, Tokyo, Japan) was performed according to the manufacturer's instruction. RNA was separated with 8 M urea-9% PAGE and ^32^P signals were detected using a Fuji BAS-2500 bio image analyzer (Fujifilm, Tokyo, Japan). For guanylyltransferase reaction, the replication protein-purified fractions were mixed with AdoMet (100 µM), GTP (1 µM), and [α-^32^P] GTP (10 µCi in 30 µL reaction mixture) and incubated at 25°C for 30 min. Protein samples were precipitated with 10% trichloroacetic acid and dissolved in standard SDS-PAGE sample buffer, then separated with NuPAGE 3–8% Tris-acetate gel (Invitrogen).

## Supporting Information

Figure S1 Effects of the H81A mutation in ToMV replication protein genes on RNA 5′ capping and RdRp functions. Wild-type and H81A ToMV RNAs (1 µg) were translated in membrane-containing BYL reaction mixtures (50 µl). Top panel shows an immunoblot of translation products detected by anti-ToMV replication protein antibodies. To eliminate excess GTP, membrane fractions of BYL reaction mixtures were collected by centrifugation at 15000 xg and subjected to protein guanylation and RdRp assays. The positions of ToMV genomic RNA (gRNA), subgenomic RNA (CPsg), and RF RNA are indicated to the right. Note that ToMV genomic RNA is a substrate of host CCA tRNA nucleotidyltransferase and a part of ^32^P in ToMV genomic RNA might be introduced by this enzyme.
(EPS)

Table S1 List of primers used in this study.
(DOCX)
