# Supplementary material for: A Host Small GTP-binding Protein ARL8 Plays Crucial Roles in Tobamovirus RNA Replication
Source: PLoS Pathog. 2011 Dec 8;7(12):e1002409. doi: 10.1371/journal.ppat.1002409 (PMC3234234; doi:10.1371/journal.ppat.1002409)
Supplement: Table S1 — List of primers used in this study. (DOCX) [file ppat.1002409.s002.docx]

Table S1. Oligonucleotides used in this study

| Name | Sequence　(5’-3’) |
| --- | --- |
| #1 | GGAGGCCTGTGGGATTATATTTGTATATT |
| #2 | CCTTATAATCACCTCCGGATCCACTCTTTGATTTTGAGTG |
| #3 | ATCAAAGAGTGGATCCGGAGGTGATTATAAGGATGATGATGAT |
| #4 | GCGAATTCTCACTTTTCAAATTGAGGATG |
| #5 | CAGATCCCCAACCACCATCATCT |
| #6 | CATTCTTCCGAACTTCTGGTG |
| #7 | CTGTCTGTTGTCTCTGTTTCTA |
| #8 | CATCAACTACATACCTGAAAGCA |
| #9 | CTGCAATATTACCACAGCAAC |
| #10 | CTTCTTAATTGGCTCCGGAG |
| #11 | GAGAGATCGGTGTTTATAGTG |
| #12 | TCAGTAGCAGAAGAAAGGGTGTCATCT |
| #13 | GATCCGTGGAGGAGAATTCTC |
| #14 | GAGAGACGGAAGGCGAAAAATAA |
| #15 | GGGCTCGAGGAAGAAATGATGGTAAATGAAATAGG |
| #16 | CCAACAAACCCATATTGTGTTTGTTAGCTTGGAG |
| #17 | CAAACACAATATGGGTTTGTTGGAAGCTTTTC |
| #18 | GGCTCGAGGTTCTTCGACTTTGAATGCTTTACG |
| #19 | GGGATCGATATGGGTTTGTTGGAAGCTTTTC |
| #20 | CGCGGCGCCGATCCCATTACCGACATTTGG |
| #21 | GGTACGTATACAGTTTGTTTTTCTTAATATCTATT |
| #22 | AAGGGAGAGCTCGCTTTGGACTTCTTCGCCAGAGG |
| #23 | GGCCAAGCTTCGGTAGAGGTGTGGTCAATAA |
| #24 | GTAGCGGCCGCGAGCT |
| #25 | CGCGGCCGCTAC |
| #26 | GG**TAATACGACTCACTATA**GTATTTTTACAACAATTACCAACAACAACA |
| #27 | TGTTGTTGTTGGTAATTGTTGTAAAAATAC**TATAGTGAGTCGTATTA**CC |

Restriction sites are underlined, T7 promoter sequence is in bold.#16
